# Scaffolding the social mind: emotion recognition supports mentalizing but reinforces bias

**DOI:** 10.1093/scan/nsag039

**Published:** 2026-06-05

**Authors:** Carolina Biasiotti, Daniela Ruzzante, Federica Meconi, Jeroen Vaes

**Affiliations:** Department of Psychology and Cognitive Science, University of Trento, Corso Bettini 84, Rovereto (TN), Trentino Alto-Adige, 38068, Italy; Department of Psychology and Cognitive Science, University of Trento, Corso Bettini 84, Rovereto (TN), Trentino Alto-Adige, 38068, Italy; Department of Psychology and Cognitive Science, University of Trento, Corso Bettini 84, Rovereto (TN), Trentino Alto-Adige, 38068, Italy; Department of Psychology and Cognitive Science, University of Trento, Corso Bettini 84, Rovereto (TN), Trentino Alto-Adige, 38068, Italy

**Keywords:** mentalizing, emotion recognition, event-related potential, oddball paradigm

## Abstract

Mentalizing—inferring others’ mental states—is a core social function supported by both perceptual and contextual processes. Emotion recognition similarly unfolds in two stages: early perceptual decoding of facial features followed by context-sensitive interpretation. Here, we tested whether emotion recognition scaffolds mentalizing across these stages and whether they attenuate or amplify social bias. Participants (*N* = 34) completed a face categorization task during EEG recording, in which mentalizing was operationalized as differential neural responses to mindful (human) versus mindless (doll-like) faces, while perceptual (neutral vs. smiling) and contextual (ingroup vs. outgroup) cues were systematically manipulated. Early neural responses differentiated human from doll-like faces, indicating rapid mind detection despite perceptual similarity. Smiling expressions enhanced early neural sensitivity across both human and doll-like faces, suggesting that emotional information is extracted at early perceptual stages independently of stimulus humanity. Later EEG components revealed an asymmetry: emotional expressions selectively boosted mental state attribution for ingroup faces, reflecting the integration of contextual social information at later stages. Implicit association tests confirmed this pattern, with mind-related concepts more strongly associated with smiling ingroup faces.

## Introduction

Humans navigate the social world by interpreting both cognitive (e.g. intentions, thoughts, and reasoning) and emotional information. These aspects, referred to as “mental states,” are ascribed to others through the process of mentalizing^1^. Within this framework, emotion recognition enables individuals to identify and interpret emotional expressions, contributing to mental state attribution. Both emotion recognition and mentalizing unfold over time and involve multiple stages ranging from the perceptual encoding of stimuli to the contextual integration required for interpreting mental states. The present study examined how emotion recognition scaffolds mentalizing by carrying early perceptual information into later, context-sensitive stages of emotional mental state attribution.

Mentalizing is part of the broader class of social-cognitive processes, considered uniquely human, as it requires a capacity for meta-representation that is absent in other species (Gilead and Ochsner 2021). The primary gateway to understanding others’ mental states is the human face and its expression. Remarkably, even brief exposure (less than 100 ms) is sufficient to infer complex mental capacities from faces (Willis and Todorov 2006). Minimal visual features, such as the eyes (Looser and Wheatley 2010), facial structure ratios (Sessa and Meconi 2015, Deska and Hugenberg 2017, Ruzzante and Vaes 2021) or general facial processing patterns (e.g. configural processing, Hugenberg et al. 2010, Fincher et al. 2024) can quickly trigger judgments about a wide range of mental capacities. These rapid inferences occur alongside other cognitive mechanisms involved in the decoding of facial expressions, serving as an early foundational process that supports and scaffolds mentalizing. For example, emotion recognition plays a pivotal role in interpreting the dynamic configurations of facial muscles, which provide immediate nonverbal cues to others’ emotional states, emerging as early as 200 ms after the stimulus onset (Calvo and Nummenmaa 2016, Cassidy et al. 2022, Yang et al. 2023).

A single perceptual cue drives information about a mental state, but context provides a crucial interpretative framework for decoding social signals and attributing mental states. Prior experiences (Carroll and Russell 1996, Frith and Frith 2006, Meconi et al. 2021), mental schemas (Barrett and Kensinger 2010, Aviezer et al. 2012), expectations, and biases all shape and refine emotional and mental state representations, enhancing their complexity and accuracy. Group membership and the social identification process further contribute to contextual modulation. People are generally more reluctant to attribute complex mental capacities to outgroup members (Blascovich et al. 2001, Hackel et al. 2014) and often distinguish between ingroups and outgroups in the attribution of unique human emotions (Leyens et al. 2000, 2001), even when group distinctions are based on minimal and arbitrary cues, as demonstrated by the minimal group paradigm (Tajfel et al. 1971, Demoulin et al. 2009). Specifically, the ingroup overexclusion effect (IOE; Yzerbyt et al. 1995) suggests that ingroup faces are protected from being mistaken as mindless. In categorization tasks, participants are more cautious and less likely to misattribute ingroup members as they lack a mind, especially when compared with outgroup members.

In light of these considerations, mentalizing appears to be initiated by low-level perceptual cues that are subsequently integrated with contextual information to refine the understanding of others’ mental states. This two-stage process was empirically modeled by Ruzzante and Vaes (2021, 2023, 2024), who outlined a neural timeline of mentalizing characterized by early perceptual processing, indexed by the N170 ERP component, and later higher-order mechanisms, marked by the P3 component. In this study, we extend this model by proposing emotion as the primary mental content for attribution. In support of this choice, emotion recognition follows a parallel neurocognitive architecture: its early stage is characterized by the holistic processing of facial stimuli, as indexed by ERP components such as N1, P1, and N170 (Eimer and Holmes 2002, Bar-Haim et al. 2007), whereas its later stages involve contextual integration and conceptual appraisal, reflected in the modulation of the P3 (Aviezer et al. 2008, Yang et al. 2023). The temporal alignment between these two independent processes allows emotion recognition to effectively scaffold the mentalizing sequence. Neuroscientific research has further clarified the sequential stages linking perception and contextual elaboration in facial and emotional processing. Specifically, the P2 component has been linked to affective processing and selective allocation of attention to emotionally and socially salient stimuli, indicating the presence of an early vigilance mechanism (Schupp et al. 2004, Carretié et al. 2007). Subsequently, the N2 component is thought to reflect a more advanced stage of discriminative processing. This stage is involved in the initial encoding of affective patterns, as well as in the evaluation of social relevance, such as familiarity and group membership (Ito and Urland 2003, Huang and Luo 2006).

Building on this framework, the present study investigated the role of emotion recognition in scaffolding the mentalizing process. To this end, we integrated both perceptual and contextual mechanisms by employing the paradigm developed by Ruzzante and Vaes (2021), which allows for the simultaneous manipulation and isolation of these factors. In the first stage, we examine how two sensory dimensions—humanness (human vs. doll-like faces) and emotional expression (neutral vs. smiling)—contribute to distinguishing mindful from mindless stimuli—i.e. the earliest stage at which neural responses differentiate the presence of a mind. In the second stage, the degree of neural differentiation between these stimuli serves as an index of mind attribution, which we assessed in relation to contextual cues, specifically group membership.

We examined whether emotional expressions modulate mental state attributions across group membership, enhancing ingroup attribution or mitigating differences toward outgroup faces. We also investigated intermediate stages of this process to clarify how emotion recognition functionally supports mentalizing. Overall, this study clarifies how emotional cues provide the representational content necessary to trigger mentalizing across both early perceptual and later contextual stages.

## Present research

To test our hypotheses, we manipulated both perceptual and contextual information. Smiling faces were used as perceptual cues for happiness, an emotion selected because it is consistently recognized with high accuracy and low ambiguity (Calvo and Beltrán 2013). Contextual cues were manipulated by assigning faces to either an ingroup or an outgroup based on national identity. The ingroup consisted of Italians, while the outgroup comprised Romanians, the largest immigrant group currently residing in Italy (Istituto Nazionale di Statistica [ISTAT] 2025). To control for the other-race effect (i.e. the tendency to recognize faces of one’s own ethnicity more accurately than those of other ethnicities; Jiang et al. (2023)), all the facial stimuli were selected from the same ethnic background. This ensured that there were no perceptual clues to distinguish between the two groups. Faces were divided into two sets with group membership counterbalanced across participants. We employed an oddball paradigm in which frequent stimuli were real human faces—either in-group or out-group—occasionally interrupted by doll-like faces. This design allowed us to investigate how the perception of emotional expressions and group membership is influenced by the presence or absence of an attributable mind. Specifically, by using perceptually similar faces that differed only in their perceived capacity to possess a mind (real human faces vs. doll-like faces), we were able to disentangle the contribution of perceptual features (e.g. emotional expression) from contextual cues (e.g. group membership) in the process of mind attribution. Additionally, we developed the Implicit Mind Attribution Test (IMAT), a modified version of the Implicit Association Test (IAT; Greenwald et al. 1998) that assesses participants’ humanization of faces through their implicit associations between the same in-group and out-group human faces used in the main task and attributes related to the mind or body. The current design was informed by an initial pilot study (see SOM, section 1), which highlighted key limitations of the original procedure, including the use of a minimal group paradigm and fixed emotion blocks. These insights guided adjustments to enhance the experimental manipulation and stimulus variability.

We expected the N170 component to represent the first stage when mindful human and mindless doll-like stimuli are distinguished. This component has been repeatedly associated with the early recognition of real human faces (Wheatley et al. 2011, Luck 2014). This effect should occur regardless of the emotional expression or group membership of the faces. Instead, we expected emotional information to modulate P3 amplitude differently depending on whether the face belonged to an ingroup or outgroup member. The amplitude of the P3 component in the oddball paradigm is influenced by stimulus frequency, with greater activation for less frequent stimuli. Moreover, this effect depends on the degree to which the infrequent stimulus is processed as distinct relative to the frequent stimuli. Therefore, we anticipated a higher P3 amplitude for doll-like faces, both infrequent and mindless, compared to real human faces, which are frequent and mindful, especially when these doll-like faces represent ingroup members. Specifically, we derived the difference in processing between human and doll-like faces as an index of mentalizing. A larger distance between the two components indicates that the two types of face are perceived as more distinct, reflecting different sensitivities to the difference between mindful entities and those without a mind. Conversely, a smaller distance reflects greater similarity in the processing of human and doll-like faces, suggesting a reduced attribution of mental complexity to human stimuli. Furthermore, we examined how this perceptual distance varies as a function of group membership and emotional expression displayed by faces. Two possible outcomes are predicted based on the existing literature and the design of our stimuli. First, consistent with well-established findings on ingroup bias, we expected a larger perceptual difference (i.e. a greater distinction between human and doll-like faces) for ingroup members compared to outgroup members, particularly in the emotional condition. This pattern reflects greater sensitivity and stronger attribution of mental states to ingroup faces. Alternatively, given that the stimuli are perceptually matched and differ only by a contextual label of group membership, we hypothesized that explicit recognition of emotional expressions (e.g. a smile) may override or neutralize these social biases. In this case, the group difference in perceptual distance between human and doll-like faces would diminish or disappear in the emotional condition, whereas it would persist in the neutral condition. This hypothesis aligns with research showing that sharing positive emotions can reduce intergroup bias and promote greater social inclusion (Paolini et al. 2021). Finally, consistent with our interpretation of the results as reflecting mentalizing processes, we hypothesized a relationship between the neural measure of the P3 component and the behavioral measure represented by the IMAT index. Specifically, reflecting the expected pattern on P3, we hypothesized that IMAT captures a stronger implicit association of mind-related attributes with ingroup faces and body-related attributes with outgroup faces, regardless of the emotional expression of the targets.

## Materials and methods

### Participants

Forty participants aged 19–32 (*M* = 22.5; SD = 2.72) took part in the study. All participants were Italian, had normal or corrected vision, and no history of neurological disease. Six of them were excluded because they had less than 70% of the remaining epochs for analysis. The analysis was therefore conducted on a total of 34 participants (19 females, *M*_age_ = 22.21, SD_age_ = 2.97; 15 males, *M*_age_ = 22.93, SD_age_ = 3.56). A sensitivity power analysis conducted using PANGEA for a repeated-measures ANOVA (2 × 2 × 2 design) indicated that, with a sample size of 34, α = 0.05, and power = 0.81, the study was capable of detecting a minimum effect size of d = 0.49 (*η^2^_p_* = .057). This sample size is consistent with typical EEG research standards and previous studies that used a similar paradigm (Vaes et al. 2019, Ruzzante and Vaes 2021). Moreover, all the significant effects reported below have a larger effect size than this minimal threshold.

### Stimuli

**Figure 1:**
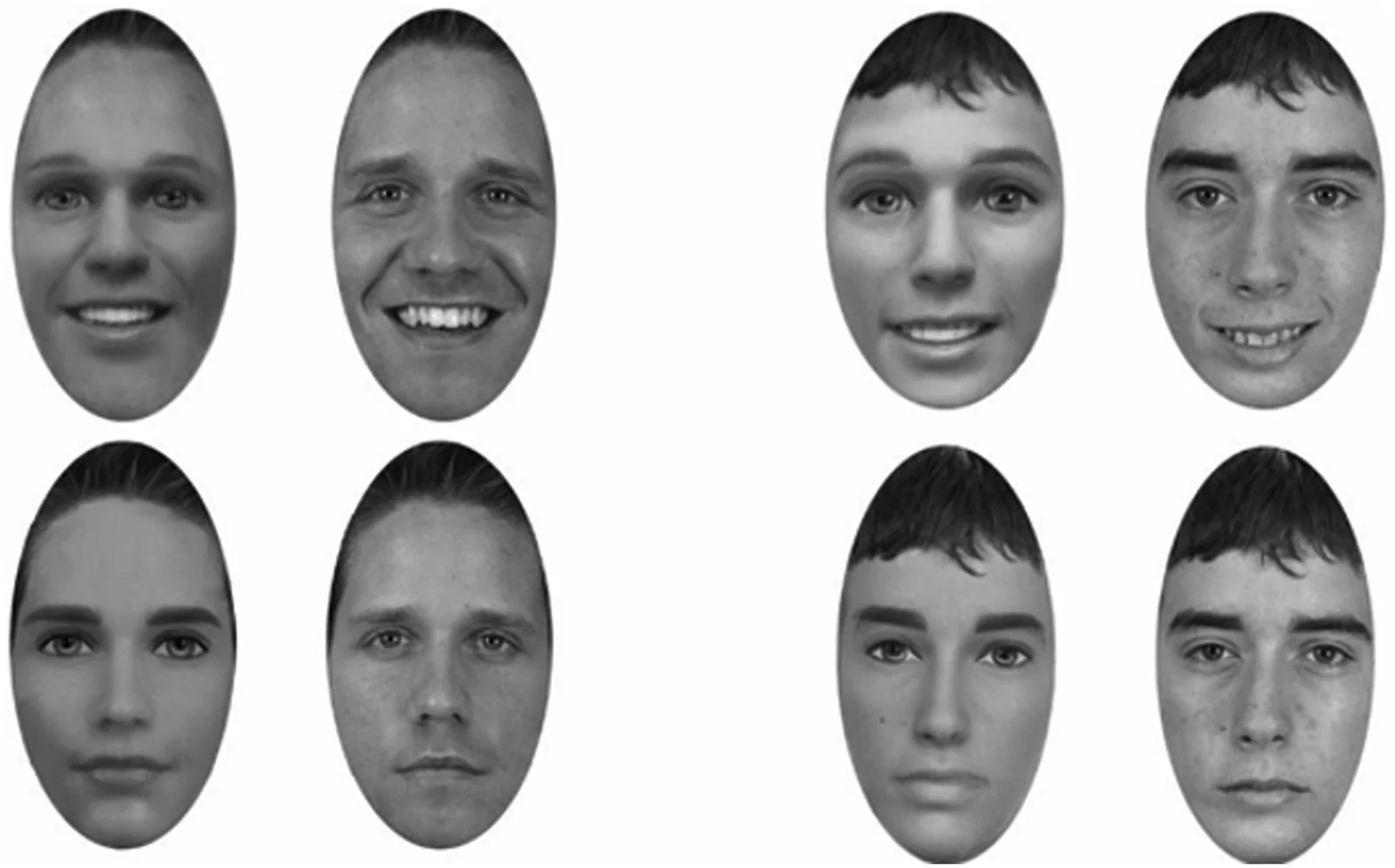
Examples of stimuli used in the experiment.

We selected 30 Caucasian male faces from the Chicago Face Database (Ma et al. 2015) and assigned them to two fictitious groups. Each face had both neutral and happy expressions, for a total of 60 images. Faces were controlled for differences in various social and perceptual attributes such as ethnic prototypicality, expression intensity, and gender (see SOM, section 2). Doll-like versions were created using Squitzmorph (Squizzmorph [Computer software], 2009), blending 30% of the human face with 70% of the doll face. To enhance artificiality, a plastic-like skin texture was applied using Photoshop (Adobe Systems Incorporated, 2022). In total, 120 images were generated: 30 neutral human faces, 30 smiling human faces, 30 neutral doll-like faces, and 30 smiling doll-like faces (see Fig. 1 for examples).

We balanced the luminance between the human and doll-like faces, converting each image to grayscale, and equalizing the luminance using MATLAB (MathWorks 2022). Each stimulus was pre-tested to ensure that the human and doll-like faces of both fictitious groups were judged with equal ease and that emotions were recognized appropriately and with the same ease between neutral and emotional faces. The pre-test was submitted to 30 participants who did not participate in the actual experiment (see SOM, section 2.1).

## Procedure

### Categorization task

After signing the informed consent form and preparing for the EEG recordings, participants received instructions for the experiment’s first part. During the main task, they categorized human- and doll-like faces using two keyboard keys. The visual stimuli were organized into four blocks that were randomized across the participants. Two blocks displayed the faces of ingroup members and two blocks showed outgroup faces. Within each group category, emotional expressions (smiling or neutral) were randomized, resulting in four blocks: two ingroup (smiling and neutral) and two outgroup (smiling and neutral) blocks. Since all the faces depicted individuals of the same ethnicity, with no perceptual cues of group membership, each block began and ended with an on-screen prompt specifying whether the faces belonged to the ingroup or outgroup. The study used an oddball paradigm, with 80% of human faces as frequent stimuli and 20% of doll-like faces as infrequent stimuli. Each block had 270 trials, including two attention checks, for which participants were asked to recall the nationality of the last face seen, for a total of 1080 trials. Within each block, stimuli were presented in a randomized order according to predefined criteria: no consecutive infrequent stimuli, between three and five presentations of the frequent stimulus, and no consecutive repetition of the same stimulus, in either the human–human or human–doll versions. Participants were seated 60 cm from the screen, with the target images displayed 2.67° below the center of a uniform gray background. A fixation cross was positioned 1.91° above the center and displayed at the beginning of each trial for 1500 ms, remaining on-screen until the face appeared. The face stimulus then remained on the screen until the participant responded. At the conclusion of this first task, the EEG headset was removed, and participants completed the Implicit Mind Association Test (IMAT) on a laptop computer.

### IMAT

Participants completed an additional categorization task involving the same human faces as in the previous task, along with ten words related to “Mind” (personality [personalità], morality [moralità], memory [memoria], self-control [auto-controllo] and thought [pensiero]) or “Body” characteristics (tone [tonicità], muscles [muscoli], movement [movimento], agility [agilità], and strength [forza]). To indicate group membership, each face was framed with either a green or a yellow border. Color assignment to the ingroup and outgroup was counterbalanced across participants to ensure no systematic bias. The IMAT began with two practice blocks in which participants categorized only the attribute words or target images.

These practice blocks were followed by a critical block, in which either compatible or incompatible associations were measured. Compatibility/incompatibility was defined based on these hypotheses. Compatible trials were defined as those in which participants categorized ingroup faces with “Mind” words and outgroup faces with “Body” words; in incompatible trials, these pairings were reversed. The order of the two experimental blocks and all variables was counterbalanced across the participants (see SOM, section 3). The IMAT was programmed and presented using Inquisit 5 (Millisecond Software 2016). The index was then calculated by subtracting incompatible trials from compatible trials, such that positive values would reflect a stronger association between ingroup faces and mind-related words.

### EEG recording and preprocessing

The EEG signal was recorded using a 64-channel headset, with electrodes placed according to the 10-10 international system and referenced online to the left mastoid. Electrode impedances were kept below 10 kΩ, and the signal was amplified using BrainAmp and sampled at 1000 Hz. Data processing was performed using MATLAB R2021b (MathWorks, Inc, 2022) with EEGlab (Delorme and Makeig 2004) and ERPlab plugins (Lopez-Calderon and Luck 2014). EEG signals were re-referenced offline to the average of the mastoids and mastoid electrodes (M1, M2), and EOG channels were initially excluded. The signal was downsampled to 250 Hz for efficiency while preserving the temporal resolution. A 0.1–40 Hz band-pass and 50 Hz notch filter were applied. Channels identified as problematic owing to poor signal quality were detected through automated threshold-based detection. As such, epochs with amplitude values outside the predefined range of ±100 μV for each channel were marked and removed if they were identified as artifacts after visual inspection to ensure artifact-free data. Independent component analysis identified and removed ocular and muscle artifacts upon manual review, and the cleaned data were averaged per participant and condition, with a grand average created for visualization. Analyses were performed on individual averages. The mean number of retained epochs for each participant in each condition and block exceeded 70%.

### Statistical analysis and results

Neural and behavioral data were analyzed using a 2 × 2 × 2 repeated-measures ANOVA in SPSS (SPSS Statistics for Windows, version 22.0), with the following factors: humanity (doll-like face vs. human), emotion (neutral vs. similing), and group membership (ingroup vs. outgroup). For all statistical analyses, the significance threshold (α) was set at 0.05. Where applicable, pairwise comparisons were corrected for multiple comparisons using Bonferroni adjustment.

### Behavioral data

We collected reaction times (RTs) and accuracy in classifying stimuli as either human or doll-like in the oddball paradigm. The RTs and accuracy scores were averaged for each stimulus type. Participants’ accuracy was significantly influenced by humanity, *F*(1,32) = 9.60*, P =* .004, *η^2^_p_=*.23. Human faces (*M = *0.95, SD = 0.21) were categorized more accurately than doll-like faces (*M* = 0.90, SD = 0.20) were. A significant interaction between emotion and group membership also emerged, *F*(1,23) = 6.19, *P* = .003, *η^2^_p_* = .24. The difference between ingroup and outgroup categorization accuracy was greater in the neutral condition (*M*_Ingroup_ = .91, SD = .22*; M*_Outgroup_  *=* .94, SD = .02) compared than in the smiling condition (*M*_Ingroup_  *=* .91, SD = .20*; M*_Outgroup_  *=* .93, SD = .20). Moreover, a significant three-way interaction between humanity, emotion, and group membership was found, *F*(1, 33) = 6.19, *P =* .018*, η^2^_p_ =* .16. Planned contrasts revealed a significant difference between human and doll-like faces across all conditions (*F* values ≥ 17.14, *P =* .001*, η^2^_p_ ≥* .35), except for smiling in-group faces, where the difference did not reach significance after Bonferroni adjustment (*P =* .12; *M*_Human_  *=* .93, SD = .03; *M*_Doll-like_  *=* .90, SD =.02). A detailed summary of the condition means is provided in SOM Table 1. This interaction supports the vigilance hypothesis, indicating an enhanced recognition accuracy for outgroup faces, particularly when perceived humanity and emotional salience are present. No other main effects or interactions reached significance (*F*s ranged from 0.16 to 2.61, all *P >* .05).

### Electrophysiological results

Based on prior literature (e.g. Ito and Bartholow 2009, Luck and Hillyard 1994, Luck 2014), we selected electrode sites and time windows for the well-established ERP components. The ERP components were defined as follows: P1 at PO8 within the 110–160 ms window (Calvo and Nummenmaa 2016), N170 within 160–210 ms at P8 and PO8 (Conty et al. 2007, Jacques et al. 2007), P2 at Cz within 160–220 ms (Calvo et al. 2013), and N2 at Fz within 220–300 ms (Williams et al. 2006). For P3, following Vaes et al. (2019), the signal was segmented into 20 ms intervals within 300–600 ms post-stimulus. We identified the onset and offset of significant amplitude differences between human and doll-like faces, defining P3 within 360–600 ms. Since our primary manipulation involved an oddball effect between human and doll-like stimuli, we conducted a functional region-of-interest (ROI) analysis informed by visual inspection of scalp topographies (see SOM, section 4, Fig. S1) using the main effect of humanity as a localizer to guide the selection of additional electrodes that are sensitive to this contrast. This approach allowed us to determine the scalp regions that showed the strongest amplitude differences related to the humanity effect, which were then used to examine interactions with other experimental factors. By localizing the effect prior to testing the interactions, we minimized the risk of circular inference (Kriegeskorte et al. 2009). To test lateralization significance, symmetrical electrode pairs from the left hemisphere were selected, including the hemisphere as a factor in the ROI analysis (see SOM, section 4). Analyses revealed a peak response over a parietal ROI, including the electrodes Pz, POz, and P2 (Luck 2012).

**Figure 2:**
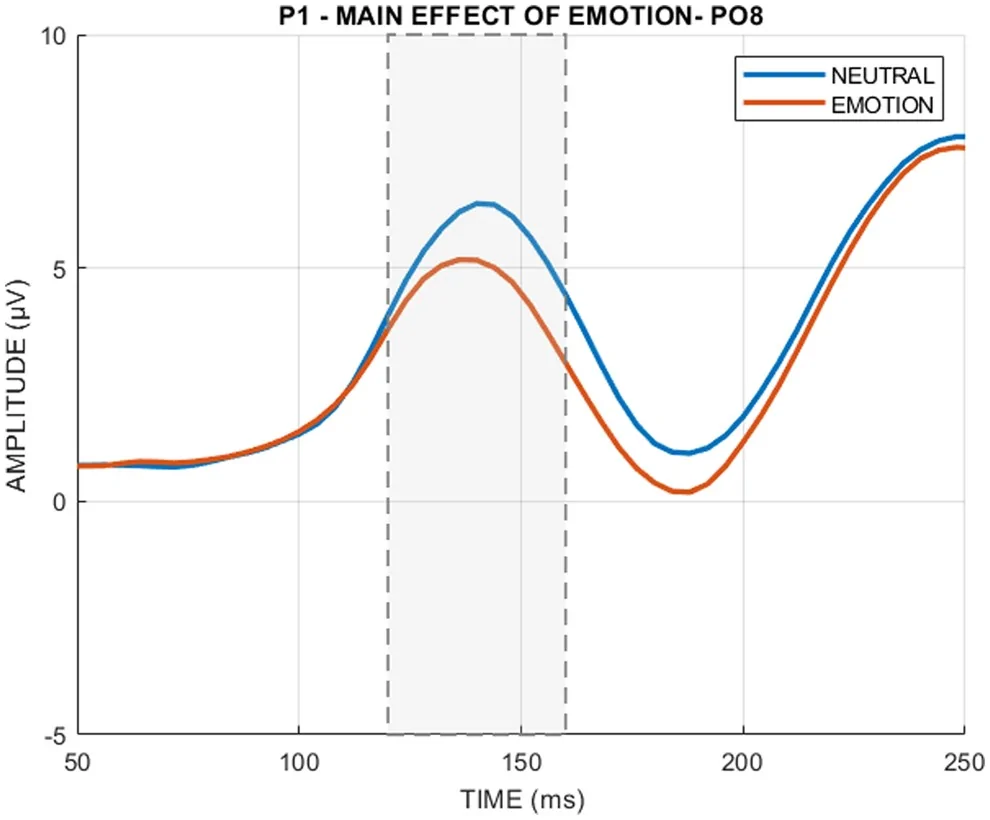
Grand-average ERP waveforms at electrode PO8 in the 160–220 ms time window, highlighting the P1 component showing a main effect of emotion.

**P1** Analysis of the P1 revealed a main effect of target emotion, *F*(1,33)=33.72*, P <* .001*, η^2^ =* .56, with larger amplitudes for smiling compared to neutral faces (see Fig. 2). This component is sensitive to early perceptual differences (Luck et al. 2000), such as facial expressions and is associated with automatic selective attention (Dennis-Tiwary et al. 2019). These results support prior findings regarding the early processing of emotional stimuli (Denefrio et al. 2017, Dennis-Tiwary et al. 2019, Schindler and Bublatzky 2020), justifying the treatment of emotional salience as a perceptual variable in subsequent mentalizing analyses. No other main effects or interactions were significant (*F*s ranged from 0.05 to 1.21, all *P* > .05).

**Figure 3:**
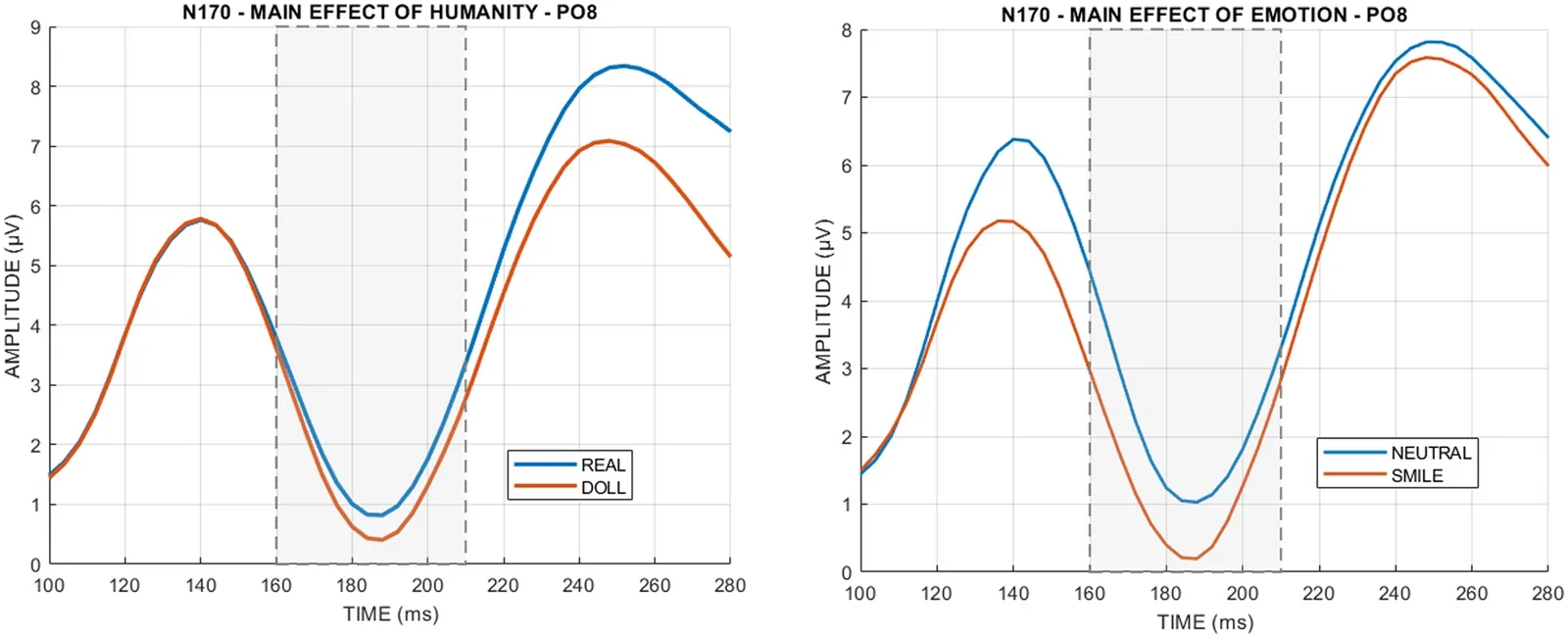
Grand average ERP waveforms at electrode PO8 in the 160–210 ms time window showing the N170 component. (a) Left panel: Humanity effect–doll-like faces evoked larger amplitudes than real faces. (b) Right panel: Emotion effect–smiling faces evoked more negative amplitudes than neutral faces.

**N170** As expected, analysis of the N170 revealed a main effect of target humanity, *F*(1,33) = 55.98, *P =* .004, *η^2^_p_ =* .23, with larger amplitudes for doll-like compared to real faces. A main effect of emotion also emerged, *F*(1,33) = 55.98, *P <* .001, *η^2^_p_ =* .63, with smiling faces eliciting larger (i.e. more negative) N170 responses than neutral faces (see Fig. 3). No other main effects or interactions reached significance (*F*s ranged from 0.06 to 1.5, all *P* > .05).

**Figure 4:**
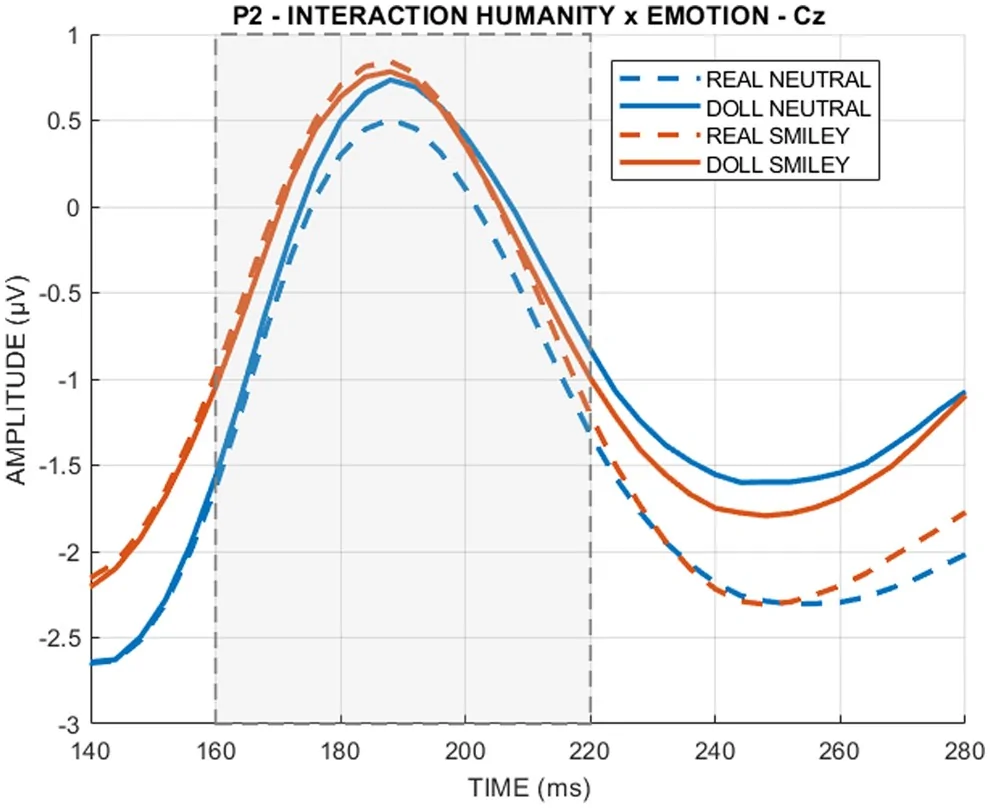
Grand-average ERP waveforms at electrode Cz in the 160–220 ms time window (P2 component), showing main effect of emotion and interaction with humanity.

**P2** Analysis of the P2 revealed a main effect of emotion, *F*(1,33) = 26.21, *P* < .001, *η^2^_p_* = .44, with larger amplitudes for emotional compared to neutral faces. A significant interaction between emotion and humanity also emerged, *F*(1,33) = 5.08, *P* = .031, *η^2^_p_* = .13. In the neutral condition, human and doll-like faces elicited different responses (*M*_Human_ = –0.64, SD =.26; *M*_Doll-like_ = –0.42, SD =.27), but this difference was not observed for emotional faces (*M*_Human_ = –0.24, SD = .27; *M*_Doll-like_= –0.25, SD = .27) (see Fig. 4). The P2 component is thought to reflect increased attentional allocation (Luck 2012), suggesting that emotional expressions enhance attention, regardless of facial realism. No other main effects or interactions reached significance (*F*s ranged from 0.003 to 4.13, all *P* > .05).

**Figure 5:**
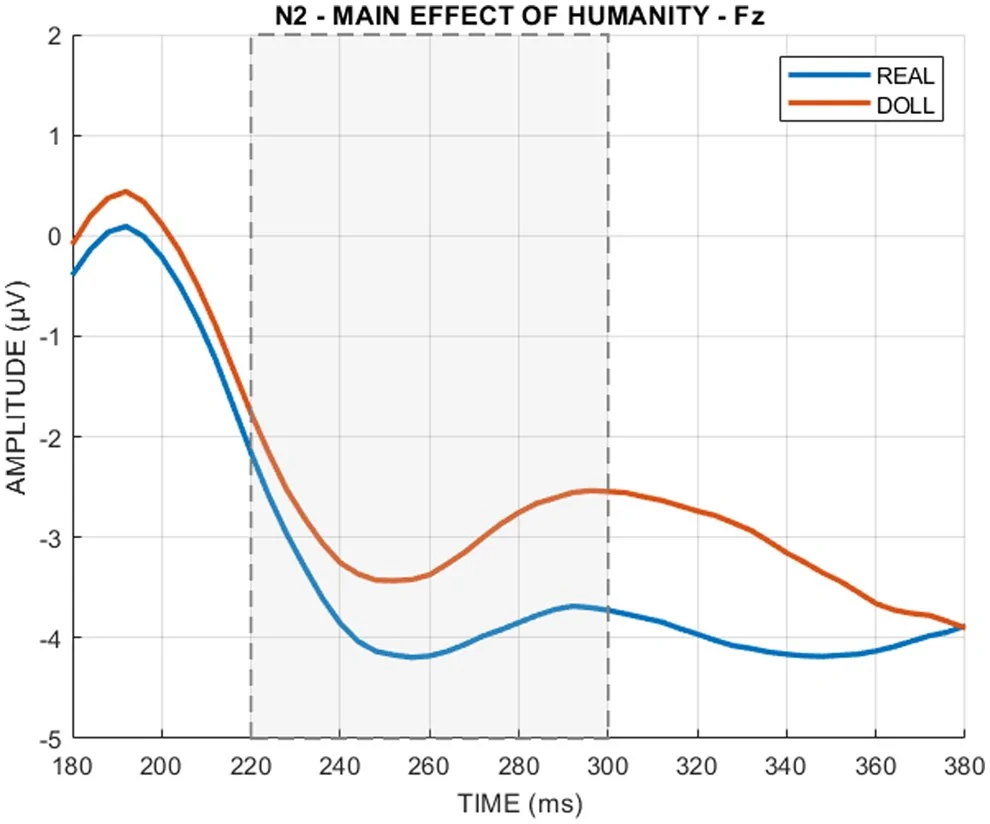
Grand-average ERP waveforms at electrode Fz in the 220–300 ms window (N2 component).

**N2** Analysis revealed a main effect of humanity, *F*(1,33) = 36.52, *P* < .001, *η^2^_p_* = .06, with a larger N2 component for real compared to doll-like faces (Fig. 5). No other main effects or interactions reached significance (*F*s ranged from 0.003 to 4.13, all *P* > .05).

**Figure 6:**
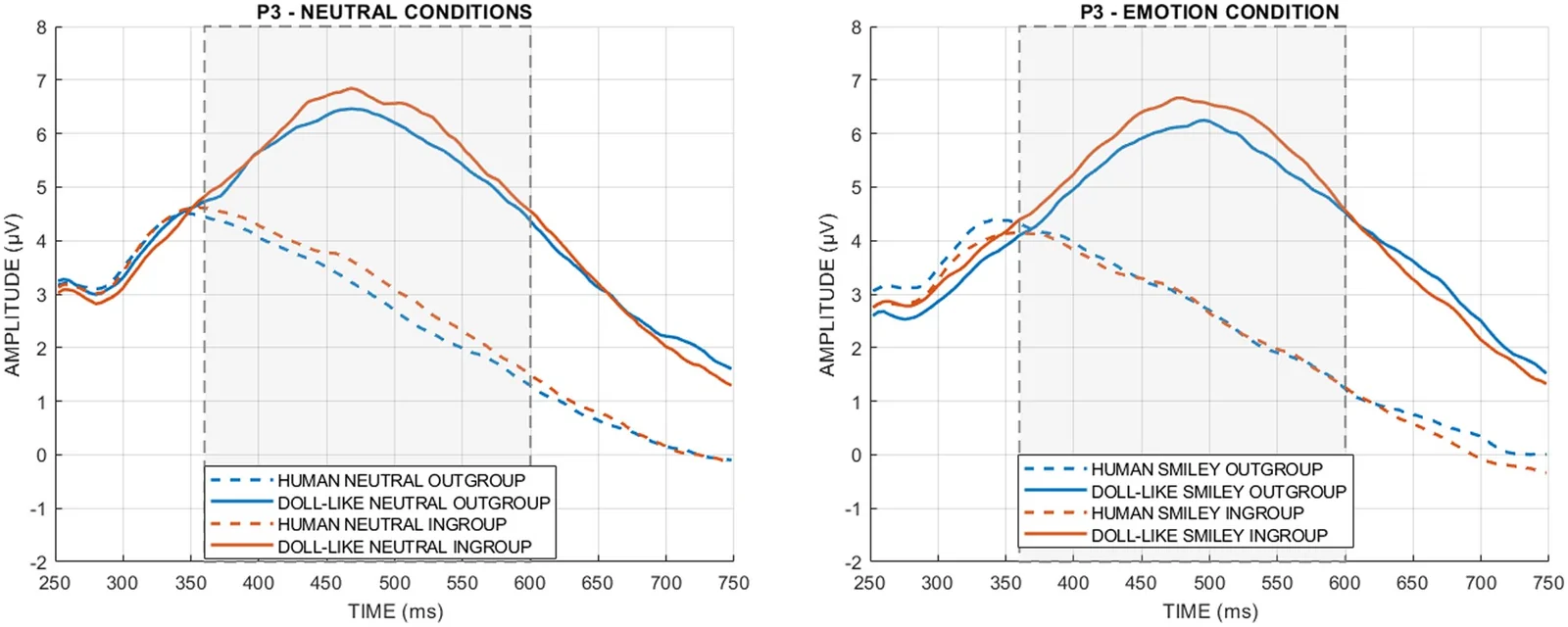
ERP mean amplitudes in the P3 region of interest (POz, Pz, P2; 360–600 ms). (a) Left panel: responses to neutral faces. (b) Right panel: responses to smiling faces.

**Figure 7:**
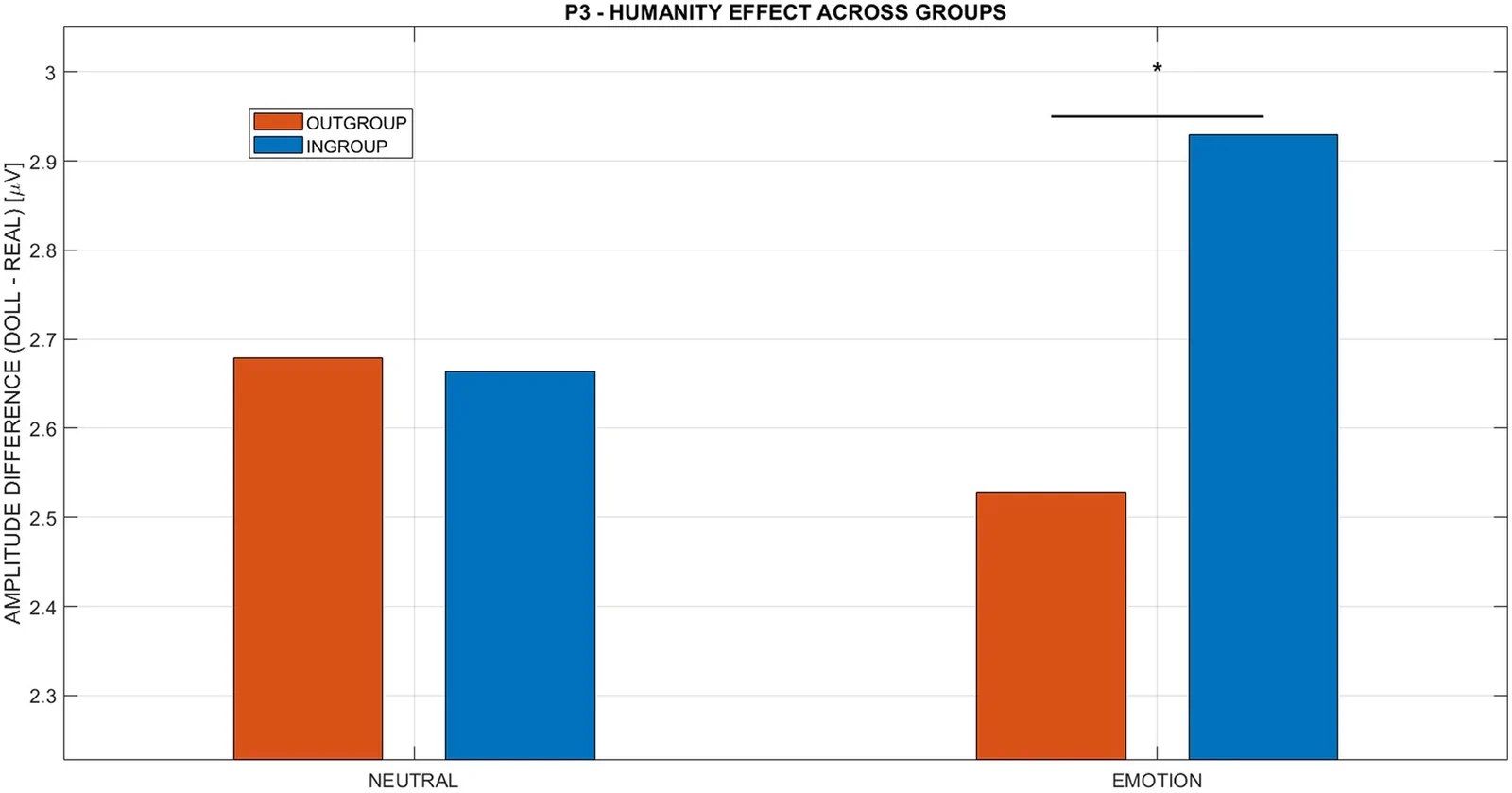
Mean amplitude differences (doll–real) in the P3 time window (360–600 ms) over the P3 region of interest (ROI: POz, Pz, P2). Bars represent the average difference for each condition, separated by group (outgroup in orange, ingroup in blue) and expression (neutral vs. emotion). A significant difference between groups was observed in the emotion condition (*P =*.033, *η^2^ =*.13).

**P3** Analysis of the P3 component revealed a significant main effect of humanity, *F*(1,33) = 58.39, *P* < .001, η^2^_p_ = .64, with larger amplitudes for doll-like (M = 5.79, SD = .59) compared to real faces (M = 2.88, SD = .38). A main effect of emotion also emerged, *F*(1,33) = 4.97, *P* = .033, η^2^_p_ =.13, with enhanced responses to neutral expressions (M = 4.43, SD = .47) compared to smiling expressions (M = 4.23, SD =.46). Critically, a three-way interaction between humanity, emotion, and group membership was found, *F*(1,33) = 4.94, *P*= .033, η^2^_p_ =.13, indicating that the P3 difference between real and doll-like faces varied depending on both expression and group. As shown in Figs 6 and 7, this modulation was strongest for ingroup members, particularly in the smiling condition. Specifically, smiling in-group doll-like faces elicited significantly larger amplitudes (M = 5.90, SD = .57) than out-group doll-like faces (M = 5.51, SD = .68), while in the neutral condition, no significant difference was observed between in-group (M = 6.01, SD = .55) and out-group (M = 5.73, SD = .65) doll-like faces. This pattern suggests that smiling expressions enhance in-group–out-group differentiation in the processing of humanized (doll-like) faces. No other main effects or interactions reached significance (*Fs* ranged from 1.34 to 2.40, all *P* > .05).

### IMAT score and ERP correlation

IMAT data were analyzed using the D-score algorithm developed for the IAT (Greenwald et al. 2003). The analysis entailed computing the mean RTs in the critical blocks, where participants categorized stimuli under either compatible or incompatible conditions.

Two separate indices were computed. *Index-E* measures implicit associations between ingroup and outgroup members, based specifically on smiling faces and their related mind–body attributions. In contrast, *Index-N* assesses the same associations but with neutral facial expressions. Higher D-scores reflect stronger implicit associations linking in-group faces with mental state concepts and outgroup faces with physical attributes. Index-E was significantly different from 0(*t*(32) = 2.92, *P* = .006, *d* = .12) and varied between .03 to .21 (*M* = .12, SD = .24), while index-N was not significantly different from 0 (*t*(32) = -.12, *P* =.90, *d*=-.01) and varied between -.11 and .10 (*M* =-.01, SD =.29). This result suggests that smiling in-group faces were more readily associated with mind-related concepts, whereas smiling out-group faces were more strongly linked to body-related concepts. Notably, no significant difference emerged between in-group and out-group neutral faces, indicating that emotional cues are crucial in modulating the perceived humanity of social targets.

Since a similar pattern was observed for neural and behavioral responses, we calculated correlations between each ERP component and the IMAT indices of both participants. Specifically, a difference score was calculated for each ERP component by subtracting the difference between the processing of human and doll-like faces in the ingroup condition from the corresponding difference in the outgroup condition (i.e. [(Ingroup_Doll-like—_Ingroup_Human_)–(Outgroup_Doll-like—_Outgroup_Human_)]). Correlations between ERPs for neutral faces and Index-N and those for smiling faces and Index-E showed no significant results (see SOM, section 5). This finding suggests that the observed effects reflect a general pattern that extends beyond individual differences.

## Discussion

This study investigated how mentalizing and emotion recognition interact to shape the attribution of emotional mental states to others. Drawing on the two-stage model of mentalizing proposed by Ruzzante and Vaes (2021, 2023), we examined the joint influence of perceptual cues (e.g. human vs. doll-like faces and emotional vs. neutral expressions) and contextual information (e.g. group membership) on the temporal unfolding of this process. We hypothesized that both humanity-related and emotion-related information would be processed early in the time course and would contribute to the initial detection of the mind. At later stages, we expected that this perceptual information, once processed, would interact with contextual higher-order information to modulate the extent to which mental states are attributed to the detected mind. Specifically, we predicted that emotional expressions would enhance mental state attributions for in-group faces. In contrast, for outgroup faces, contextual information might either suppress emotion recognition, thereby reducing mentalizing, or, alternatively, emotional expressions might override contextual bias, promoting the attribution of emotional mental states even to outgroup members.

Consistent with the two-stage model of emotional processing, P1 reflected an early stage of conditionally distinct perceptual processing. However, no specific amplification was observed for emotional stimuli, suggesting that emotional salience does not directly modulate the P1 response in this region. This is consistent with studies that attribute P1 sensitivity to perceptual, rather than emotional, properties (Batty et al. 2002, Calvo and Nummenmaa 2016).

In contrast, the distinction between human (mindful) and doll-like (mindless) stimuli emerged later in the N170 component, which is consistent with the Mind Detection stage described by Ruzzante and Vaes (2021, 2023, 2023). Furthermore, the greater activation of doll-like faces mirrors the results of other studies (Zhao et al. 2019).

Emotional influence persists in the P2 component, in which emotion interacts with humanity-related perceptual cues (Vaes et al. 2016, Meconi et al. 2018). This interaction is mediated by attention and perceptual categorization guided by distinctive visual features (Luck and Hillyard 1994, Carretié and Iglesias 1995). These attentional processes extract socially relevant information, such as race and gender categories (Derks et al. 2015, Volpert-Esmond and Bartholow 2021), overcoming perceptual differences related to simple visual aspects. Emotion, as a salient element, has attracted considerable attention, regardless of face humanity. By contrast, in the absence of emotional cues (neutral faces), this attentional advantage disappears, suggesting that attention is not automatically allocated based on face humanity alone.

Although the dominance of emotion decreased in the N2 component, this can be explained as an effect of task relevance. N2 is typically triggered by attention allocation to task-relevant stimuli (Eimer et al. 2009) explaining the observed difference in processing human versus doll-like stimuli, since the primary task was to distinguish these categories. While the absence of an emotional effect at this stage may contrast with the emotion processing and empathy literature (Sessa et al. 2014, Calvo and Nummenmaa 2016, Meconi et al. 2018), this discrepancy is plausible because emotional discrimination was not required by the task.

In the final stage of mental state attribution, indexed by the P3 component, we observe the functional synthesis of prior stages. At this level, emotion recognition is no longer a separate perceptual analysis; rather, the emotional cue is integrated within the social context to determine the extent of mentalizing. This integration is likely driven by motivational engagement (Amodio and Devine 2006). Consistent with this, group differences emerged only in the emotional condition, reflecting the fact that intergroup bias depends on affective and motivational engagement rather than automatic categorization. Neutral faces may lack sufficient affective salience to elicit such bias, whereas emotional expressions provide the conditions for its emergence (Amodio et al. 2003). Specifically, emotional expressions in ingroup members enhanced the differentiation between mindful and mindless entities, which is consistent with our hypothesis on the IOE. This suggests that for the ingroup, the emotional signal (the smile) was successfully integrated as a gateway to trigger a deeper attribution of mental states. This reinforces mentalizing when social motivations (e.g. group affiliation) align with emotional signals. In contrast, for outgroup faces, emotional expressions were perceptually recognized (as evidenced by early ERP components) but did not modulate mind attribution at the later stage of the P3; consequently, neural responses to human and doll-like faces remained similar across conditions. This implies that emotion recognition, when decoupled from an ingroup social context, is insufficient to scaffold the transition from simple perception to full mentalizing. In other words, emotion recognition supports, but does not override, social biases. Overall, these findings indicate that the neural differentiation between real and artificial agents, reflected in the P3 amplitude, is dynamic rather than fixed, shaped by the interaction of emotional and contextual cues. Behavioral results from the IMAT support this, showing stronger associations between mental terms and ingroup faces only in the emotional condition. In light of these outcomes, the electrophysiological results are consistent with the extensive literature on mental attributions in intergroup contexts, which consistently reveals a tendency to favor in-group members over out-group members. Importantly, our results extend this work by demonstrating that emotion recognition modulates this bias, enhancing mentalizing only when contextual conditions are allowed. This integration reveals an intricate, stage-dependent architecture where early perceptual cues scaffold—but do not override—context-driven mind attributions. These findings should be interpreted in light of some limitations. The emotional manipulation was restricted to happy expressions, limiting generalizability to positive affect; future work should test emotions with different valence (e.g. anger, fear). The absence of explicit measures of group-related attitudes prevents assessing how individual expectations may have influenced mind attribution, particularly for outgroup faces. Future studies could also move beyond the ERP paradigm to examine the spatiotemporal dynamics of mentalizing under systematically varied social contexts.

## Conclusion

This study investigated the role of emotion recognition as a scaffolding mechanism in the process of mentalizing. Specifically, using humanness as an index of mental state attribution, we compared mindful and mindless entities within an oddball paradigm under conditions in which they either expressed an emotional state (smiling) or remained neutral.

Our results showed that, initially, the mind was reliably detected in human faces, regardless of their emotional expression. However, at later stages, mental state attribution was modulated by contextual information, specifically group membership, and its interaction with emotional expression. Notably, the distinction between mindful and mindless entities was enhanced in the ingroup condition with emotional expressions, whereas in the outgroup condition, this distinction was smaller and not affected by emotional cues. These findings show that emotion recognition supports, but does not override, social biases in mentalizing; instead, emotional signals are filtered by context to modulate the extent of emotional mental state attributions, reinforcing ingroup bias in social cognition.

## Supplementary Material

nsag039_Supplementary_Data

## Data Availability

All data supporting the findings of this study are included in the article and its Supplementary Materials.

## Footnotes

1 Following the recent expert consensus (Quesque et al. 2024), we adopt “mentalizing” as the most generic and appropriate term to describe the ability to attribute mental states—including emotions—to others. We deliberately avoid more specific labels, such as “affective mentalizing,” to maintain conceptual clarity and avoid overlap with adjacent constructs such as empathy.
