# Supplementary material for: Scaffolding the social mind: emotion recognition supports mentalizing but reinforces bias
Source: Soc Cogn Affect Neurosci. 2026 Jun 5;21(1):nsag039. doi: 10.1093/scan/nsag039 (PMC13427767; doi:10.1093/scan/nsag039)
Supplement: nsag039_Supplementary_Data [file nsag039_supplementary_data.docx]

### **1. Overview of the Preliminary Study**

This preliminary study was conducted on a sample of 16 university students to test the main hypotheses and assess the feasibility of the experimental protocol. Although it was initially intended as a full experimental run, the results revealed several methodological limitations that informed important changes to the final design. The study employed the same facial stimuli used in the main experiment, but organized them into four blocks based on group membership (ingroup vs. outgroup) and emotional expression (neutral vs. smiling). Group membership was manipulated using the Minimal Group Paradigm (Tajfel et al., 1971), operationalized through the Numerical Estimation Style Test (NEST) (Ratner & Amodio, 2012). Faces were labelled as “overestimators” or “underestimators” using a colored frame (green, RGB: 116, 181, 53 vs. yellow, RGB: 188, 207, 66). These colors were chosen for their perceptual distinctiveness while maintaining equal luminance.

### **1.2 Key Results**

Although the paradigm was well implemented, the results highlighted some limitations:

- No effect of emotional expression emerged, possibly due to participants being exposed to a single emotion type per block (neutral or smiling), which may have reduced salience or contrast.
- Faces were correctly categorized, but the emotion recognition measures (intensity and congruence) showed differences between doll-like and human faces, suggesting a potential confound.
- No critical effects were found regarding group membership, suggesting the Minimal Group manipulation was too weak in the context of repeated exposure to similar stimuli.

### **1.3 Changes to the Final Design**

Based on the limitations identified in this preliminary study, we introduced three major changes to the final protocol:

1. Stronger social categorization: We replaced the minimal group paradigm with nationality-based labeling, to provide a more salient and ecologically valid social context.
2. Emotion randomization within blocks: Instead of separating blocks by emotion, neutral and smiling faces were randomized within each group-membership block. This was done to enhance the contrast and salience of emotional expressions.
3. Expanded IAT task: In the pilot, the Implicit Association Test (IAT) included only neutral expressions; in the final study, we included both neutral and smiling faces to better capture differential associations by emotion.

#### **2. Creation of the Stimuli**

Faces were carefully controlled for social attributions such as fearfulness, anger, attractiveness, babyfacedness, disgust, ethnic prototypicality, happiness, dominance, gender, surprise, sadness, trustworthiness, fitness, threat, unusualness, luminance, and Facial Width-to-Height Ratio (FWHR). No significant differences between the two groups of faces were found on any of these dimensions.

#### **2.1 Pre-test**

A pre-test was conducted to validate the stimuli in terms of recognizability, emotional clarity, and group assignment. Thirty participants (not involved in the main experiment) completed the pre-test.

**2.2 Procedure:** In the first part, participants categorized each face as either *human* or *doll-like* by pressing a key as quickly and accurately as possible. In the second part, they responded to a series of questions: (1) identifying the expressed emotion; (2) rating how well that emotion matched the expression; and (3) judging the intensity of the expression on a 7-point scale (1 = “Not at all”, 7 = “Completely”).

The task was programmed using Inquisit 5 and data were analyzed with SPSS (version 25, IBM Corp.).

#### **2.3 Results Summary**

A 2 (Humanity: Human, Doll-like) × 2 (Emotion: Neutral, Smiling) repeated-measures ANOVA on categorization accuracy revealed a main effect of Humanity*, F(1,31) = 5.17, p = .030, ƞ²ₚ = .14*. Doll-like faces (M = .94, SD = .07) were categorized more accurately than human faces (M = .92, SD = .09). No significant main effect of Emotion, *(F(1,31) = 1.27, p = .26,)* or interaction*, (F(1,31) = 0.61, p = .80),* emerged. Reaction time (RT) analyses also yielded no significant effects for Humanity, *(F = .39, p = .53),* *Emotion, (F = 1.93, p = .17)*, or their interaction, *(F = 1.72, p = .19)*.

For emotional judgments, a 2 (Humanity) × 2 (Expression) ANOVA confirmed no main effect of Humanity on emotion recognition accuracy, *(F = 3.51, p = .070),* but significant effects for perceived emotional adequacy*, F(1,31) = 58.13, p < .001, ƞ²ₚ = .65*, and intensity, *F(1,31) = 93.06, p < .001, ƞ²ₚ = .75*. Human faces were perceived as expressing emotions more adequately (M = 5.80, SD = .79) and more intensely (M = 5.35, SD = .90) than doll-like faces (M = 4.72, SD = 1.23; M = 4.26, SD = 1.24, respectively).

Despite these differences, our main objective was to test whether ingroup vs. outgroup emotion recognition modulated mentalization. Thus, we considered these differences in adequacy and intensity as non-critical for the main analyses.

#### **2.4 Stimuli Grouping and List Matching**

To define group membership (ingroup vs. outgroup), faces were divided into two balanced lists (A and B). We ensured that the stimuli were matched across lists in terms of emotion recognition and categorization performance.

**List A:** A significant effect of Humanity was found for categorization accuracy, *F(1,31) = 6.63, p = .015, ƞ²ₚ = .17*, with doll.like faces (M = .946, SD = .013) categorized more accurately than human faces (M = .916, SD = .017). No effects emerged for emotion, *F = .57, p = .45*, or the interaction, *F* = .83, *p* = .77. Reaction times showed no effects of Humanity (*F* = .28, *p* = .59), Emotion (*F* = 3.28, *p* = .08), or interaction (*F* = .20, *p* = .65).

**List B:** No significant effects were found for Humanity (*F* = .77, *p* = .38), Emotion (*F* = 2.09, *p* = .15), or their interaction (*F* = .00, *p* = 1.00). Reaction time analyses were likewise non-significant.

**Between-list Comparison:** No significant differences were found in accuracy between human faces from lists A and B, (*F* = .00, *p* = 1.00), or between doll-like faces, (*F* = .33, *p* = .57). Reaction times were also comparable across lists, *(F = .74, p = .40)*. One small but statistically significant interaction between List and Humanity on RTs, *(F = 2.28, p < .001)* showed that participants responded slightly faster to human faces in list A (M = 599.59 ms) than in list B (M = 591.63 ms). Given the limited magnitude and lack of relevance to the ingroup/outgroup manipulation, this effect was not considered problematic.

**3. IMAT procedure**

| **Block** | **Type of Judgment** | **Instructions** | **N. Trials** |  |
| --- | --- | --- | --- | --- |
| **1** | **Ingroup/Outgroup faces discrimination (Practice)** | **Please, carefully observe each face paired with its category and press "D" for outgroup and "K" for ingroup faces** | **20** |  |
| **2** | **Word discrimination (Practice)** | **Press "D" to categorize body-related words, "K" to categorize mind-related words** | **20** |  |
| **3** | **Ingroup/Outgroup faces discrimination (Practice, smiling faces)** | **Please, carefully observe each face paired with its category and press "K" for outgroup and "D" for ingroup faces** | **20** |  |
| **4** | **Compatible categorization (Test, smiling faces)** | **Press "D" for body-related words and outgroup faces; "K" for mind-related words and ingroup faces** | **44** |  |
| **5** | **Compatible categorization (Test, smiling faces)** | ***Same as above*** | **44** |  |
| **6** | **Instruction screen (switch to incompatible blocks)** | **—** | **—** |  |
| **7** | **Incompatible categorization (Test, smiling faces)** | **Press "D" for body-related words and ingroup faces; "K" for mind-related words and outgroup faces** | **44** |  |
| **8** | **Incompatible categorization (Test, smiling faces)** | ***Same as above*** | **44** |  |
| **9** | **Instruction screen (switch to neutral face blocks)** | **—** | **—** |  |
| **10** | **Ingroup/Outgroup faces discrimination (Practice, neutral faces)** | **Please, carefully observe each face paired with its category and press "K" for outgroup and "D" for ingroup faces** | **20** |  |
| **11** | **Incompatible categorization (Test, neutral faces)** | **Press "D" for body-related words and ingroup faces; "K" for mind-related words and outgroup faces** | **44** |  |
| **12** | **Incompatible categorization (Test, neutral faces)** | ***Same as above*** | **44** |  |
| **13** | **Instruction screen (switch to compatible blocks)** | **—** | **—** |  |
| **14** | **Compatible categorization (Test, neutral faces)** | **Press "D" for body-related words and outgroup faces; "K" for mind-related words and ingroup faces** | **44** |  |
| **15** | **Compatible categorization (Test, neutral faces)** | ***Same as above*** | **44** |  |

**4. ROI Definition Justification**4.1 ROI and Lateralization Analyses

Prior to defining the posterior region of interest (ROI), we examined potential hemispheric lateralization effects. We conducted a repeated-measures ANOVA including the factor Hemisphere (Left vs. Right), using pairs of laterally symmetric electrodes. The analysis revealed a significant main effect of Hemisphere, *F(1, 33) = 19.34, p < .001, ƞ²ₚ = .37*, with stronger responses over the right hemisphere (M = 4.33, SD = 0.52) compared to the left (M = 3.92, SD = 0.43).

Moreover, a significant Hemisphere × Humanity interaction emerged, *F(1, 33) = 8.23, p < .001, ƞ²ₚ = .46*. Bonferroni-corrected pairwise comparisons confirmed greater right-hemisphere activity for both conditions, with a stronger lateralization for Doll-like faces (M = 0.656, p < .001) compared to Real faces (M = 0.164, p = .047) (see Table S1).

Given this pattern of lateralized activation, all subsequent ROI analyses were restricted to the right hemisphere.

4.2 Right-hemisphere Posterior ROI Definition

To determine whether a group of right posterior electrodes could be meaningfully combined into a single ROI, we performed a 2 (Humanity: Real, Doll) × 3 (Channel: Pz, POz, P2) repeated-measures ANOVA. This analysis tested for an interaction between Channel and Humanity. The interaction was not statistically significant, *F(1, 32) = 1.69, p = .20, ƞ²ₚ = .10*, indicating that the effect of Humanity was consistent across the three channels. Therefore, we averaged activity across Pz, POz, and P2 (right hemisphere) to compute a posterior ROI for subsequent analyses.

Figure_S1

Table_S1

Recognition accuracy as a function of group membership, emotional expression, and face type. Recognition performance was generally higher for outgroup faces than ingroup faces, despite the perceptual similarity of the stimuli, consistent with the *vigilance hypothesis* (Allport & Kramer, 1946), which posits increased attentional allocation toward potentially threatening or socially salient outgroup members. Notably, this outgroup advantage was absent in the condition involving doll-like faces with neutral expressions, suggesting that both perceived humanity and emotional salience modulate the effect.

5. **Correlations between ERP Differentials (P3) and Implicit Mind Association Task (IMAT)**

We examined whether differential neural responses of the P3 component were associated with implicit bias scores derived from the IMAT.

The correlation between the P3 differential for smiling faces and the IMAT index for the smiling condition was not significant, *r*(31) = –0.039, *p* = .831.

Similarly, the correlation between the P3 differential for neutral faces and the IAT index for the neutral condition (did not reach significance, *r*(31) = .184, *p* = .306.

**Bibliography**Allport GW, Kramer BM. Some roots of prejudice. The Journal of Psychology [Internet]. 1946 Jul 1;22(1):9–39. Available from: <https://doi.org/10.1080/00223980.1946.9917293>

Ratner KG, Amodio DM. Seeing “us vs. them”: Minimal group effects on the neural encoding of faces. Journal of Experimental Social Psychology [Internet]. 2012 Nov 21;49(2):298–301. Available from: https://doi.org/10.1016/j.jesp.2012.10.017

Tajfel H, Billig MG, Bundy RP, Flament C. Social categorization and intergroup behaviour. European Journal of Social Psychology [Internet]. 1971 Apr 1;1(2):149–78. Available from: https://doi.org/10.1002/ejsp.2420010202

**Figure legend**
**Figure S1** Topographical distribution of the main effect of humanity, used to define ROIs sensitive to the human–doll-like contrast.
